# Germline Genetic Testing and Survival Outcomes Among Children With Rhabdomyosarcoma: A Report From the Children’s Oncology Group

**DOI:** 10.1001/jamanetworkopen.2024.4170

**Published:** 2024-03-28

**Authors:** Bailey A. Martin-Giacalone, He Li, Michael E. Scheurer, Dana L. Casey, Shannon Dugan-Perez, Deborah A. Marquez-Do, Donna Muzny, Richard A. Gibbs, Donald A. Barkauskas, David Hall, Douglas R. Stewart, Joshua D. Schiffman, Matthew T. McEvoy, Javed Khan, David Malkin, Corinne M. Linardic, Brian D. Crompton, Jack F. Shern, Stephen X. Skapek, Rajkumar Venkatramani, Douglas S. Hawkins, Aniko Sabo, Sharon E. Plon, Philip J. Lupo

**Affiliations:** 1 Division of Public Health Sciences, Department of Surgery, Washington University School of Medicine in St. Louis, St. Louis, Missouri; 2 Department of Pediatrics, Section of Hematology-Oncology, Baylor College of Medicine, Houston, Texas; 3 Human Genome Sequencing Center, Baylor College of Medicine, Houston, Texas; 4 Dan L. Duncan Comprehensive Cancer Center, Baylor College of Medicine, Houston, Texas; 5 Department of Radiation Oncology, University of North Carolina at Chapel Hill School of Medicine, Chapel Hill; 6 Department of Molecular and Human Genetics, Baylor College of Medicine, Houston, Texas; 7 Department of Population and Public Health Sciences, Keck School of Medicine of University of Southern California, Los Angeles; 8 QuadW Childhood Sarcoma Biostatistics and Annotation Office at the Children’s Oncology Group, Monrovia, California; 9 Clinical Genetics Branch, Division of Cancer Epidemiology and Genetics, National Cancer Institute, National Institutes of Health, Rockville, Maryland; 10 Department of Pediatrics, Huntsman Cancer Institute, University of Utah, Salt Lake City; 11 Department of Oncological Sciences, Huntsman Cancer Institute, University of Utah, Salt Lake City; 12 Oncogenomics Section, Genetics Branch, Center for Cancer Research, National Cancer Institute, National Institutes of Health, Bethesda, Maryland; 13 Division of Haematology-Oncology, Department of Paediatrics, The Hospital for Sick Children, University of Toronto, Toronto, Ontario, Canada; 14 Department of Pediatrics, Duke University School of Medicine, Durham, North Carolina; 15 Department of Pharmacology and Cancer Biology, Duke University School of Medicine, Durham, North Carolina; 16 Department of Pediatric Oncology, Dana-Farber/Boston Children’s Cancer and Blood Disorders Center, Harvard Medical School, Boston, Massachusetts; 17 Pediatric Oncology Branch, Center for Cancer Research, National Cancer Institute, National Institutes of Health, Bethesda, Maryland; 18 Department of Pediatrics, The University of Texas Southwestern Medical Center, Dallas; 19 Division of Hematology-Oncology, Seattle Children’s Hospital, University of Washington School of Medicine, Seattle

## Abstract

**Importance:**

Determining the impact of germline cancer-predisposition variants (CPVs) on outcomes could inform novel approaches to testing and treating children with rhabdomyosarcoma.

**Objective:**

To assess whether CPVs are associated with outcome among children with rhabdomyosarcoma.

**Design, Setting, and Participants:**

In this cohort study, data were obtained for individuals, aged 0.01-23.23 years, newly diagnosed with rhabdomyosarcoma who were treated across 171 Children’s Oncology Group sites from March 15, 1999, to December 8, 2017. Data analysis was performed from June 16, 2021, to May 15, 2023.

**Exposure:**

The presence of a CPV in 24 rhabdomyosarcoma-associated cancer–predisposition genes (CPGs) or an expanded set of 63 autosomal-dominant CPGs.

**Main Outcomes and Measures:**

Overall survival (OS) and event-free survival (EFS) were the main outcomes, using the Kaplan-Meier estimator to assess survival probabilities and the Cox proportional hazards regression model to adjust for clinical covariates. Analyses were stratified by tumor histology and the fusion status of *PAX3* or *PAX7* to the *FOXO1* gene.

**Results:**

In this study of 580 individuals with rhabdomyosarcoma, the median patient age was 5.9 years (range, 0.01-23.23 years), and the male-to-female ratio was 1.5 to 1 (351 [60.5%] male). For patients with CPVs in rhabdomyosarcoma-associated CPGs, EFS was 48.4% compared with 57.8% for patients without a CPV (*P* = .10), and OS was 53.7% compared with 65.3% for patients without a CPV (*P* = .06). After adjustment, patients with CPVs had significantly worse OS (adjusted hazard ratio [AHR], 2.49 [95% CI, 1.39-4.45]; *P* = .002), and the outcomes were not better among patients with embryonal histology (EFS: AHR, 2.25 [95% CI, 1.25-4.06]; *P* = .007]; OS: AHR, 2.83 [95% CI, 1.47-5.43]; *P* = .002]). These associations were not due to the development of a second malignant neoplasm, and importantly, patients with fusion-negative rhabdomyosarcoma who harbored a CPV had similarly inferior outcomes as patients with fusion-positive rhabdomyosarcoma without CPVs (EFS: AHR, 1.35 [95% CI, 0.71-2.59]; *P* = .37; OS: AHR, 1.71 [95% CI, 0.84-3.47]; *P* = .14). There were no significant differences in outcome by CPV status of the 63 CPG set.

**Conclusions and Relevance:**

This cohort study identified a group of patients with embryonal rhabdomyosarcoma who had a particularly poor outcome. Other important clinical findings included that individuals with *TP53* had poor outcomes independent of second malignant neoplasms and that patients with fusion-negative rhabdomyosarcoma who harbored a CPV had outcomes comparable to patients with fusion-positive rhabdomyosarcoma. These findings suggest that germline CPV testing may aid in clinical prognosis and should be considered in prospective risk-based clinical trials.

## Key Points

**Question:**

What is the association of germline cancer-predisposition variants (CPVs) with outcomes among children with rhabdomyosarcoma?

**Findings:**

In this cohort study of 580 individuals aged 0.01-23.23 years with rhabdomyosarcoma, those with CPVs in rhabdomyosarcoma-associated cancer–predisposition genes were more likely to experience adverse outcomes. Children with embryonal rhabdomyosarcoma and a CPV had outcomes comparable to children with fusion-positive rhabdomyosarcoma, a more aggressive rhabdomyosarcoma subtype, and those with *TP53* CPVs had poor outcomes independent of a second malignant neoplasm.

**Meaning:**

In this study, CPVs were associated with worse outcomes among children with rhabdomyosarcoma, suggesting that the incorporation of CPV status could inform novel risk-stratification strategies.

## Teaser

This cohort study assesses the association of germline cancer-predisposition variants with outcomes among children with rhabdomyosarcoma.

## Introduction

Rhabdomyosarcoma (RMS) is the most common childhood soft tissue sarcoma.^1^ Conventionally, RMS is classified into 2 major histological subtypes: embryonal RMS (ERMS) and alveolar RMS (ARMS). Alveolar RMS is commonly driven by the fusion of *PAX3* or *PAX7* to the *FOXO1* gene (*PAX3/7::FOXO1*; ie, fusion-positive).^2,3^ In contrast, ERMS typically lacks the canonical *PAX3/7::FOXO1* fusion (ie, fusion-negative); these tumors are typically driven by somatic mutations in the RAS signaling pathway,^4,5^ loss of heterozygosity at 11p15.5,^6^ and whole chromosomal alterations.^7^

The 5-year overall survival (OS) for RMS is 65% to 70%, and despite multimodality therapy, improving survival for children with intermediate-risk and high-risk disease has been unsuccessful.^8,9,10^ Current protocols use *PAX3/7::FOXO1* fusion status rather than histology for risk stratification because fusion-positive status is a stronger prognostic factor.^11,12^ However, germline markers are not currently used in RMS risk stratification, although the presence of germline predisposition variants does guide eligibility for other pediatric cancers (Children’s Oncology Group [COG] AAML1831, COG ACNS1833). As risk stratification for RMS is still imprecise, it is important to determine whether germline predisposition variants may be informative for risk-based RMS therapeutic protocols.

Recent studies showed through exome sequencing that approximately 7% of patients with RMS harbor a germline cancer-predisposition variant (CPV).^13,14^ Patients with ERMS were more likely to harbor a germline CPV than those with ARMS (10% vs 3.0%; *P* = .02).^13^ Kim et al^14^ further evaluated outcomes among 256 patients with RMS by germline CPV status. While they found no significant associations between the presence of a pathogenic or likely pathogenic germline variant and OS or event-free survival (EFS), their analysis was limited to patients with intermediate-risk disease, and they did not evaluate outcome by tumor histology or fusion status. Therefore, we sought to evaluate the association of CPVs with outcomes in a large, unselected cohort with RMS and assess differences in outcome across RMS subtypes.

## Methods

### Study Population

This cohort study was approved by the Institutional Review Board at Baylor College of Medicine. All patients and/or their parents or guardians gave written informed consent to participate through the COG Soft Tissue Sarcoma biobanking protocol from March 15, 1999, to December 8, 2017. Inclusion criteria included patients 50 years or younger, as individuals diagnosed with RMS are frequently treated using COG protocols; other inclusion criteria were a histologically confirmed RMS diagnosis and germline exome sequencing performed at the Human Genome Sequencing Center at Baylor College of Medicine.^13^ Self-reported race and ethnicity categories included Hispanic, non-Hispanic Black or African American, non-Hispanic White, or other non-Hispanic racial or ethnic group (including Alaska Native, Asian, or Native Hawaiian or Pacific Islander). Race and ethnicity were included in the study because there are potential differences in outcome based on this information. Additional information on data collection can be found in the eMethods in Supplement 1. This study followed the Strengthening the Reporting of Observational Studies in Epidemiology (STROBE) reporting guideline.

### Cancer-Predisposition Genes and Variants

Germline CPVs were identified as previously described.^13^ Briefly, we analyzed 24 autosomal-dominant cancer–predisposition genes (CPGs) that are linked to cancer–predisposition syndromes and that have been previously associated with RMS predisposition in multiple assessments. In a separate analysis, we expanded this gene list to include an additional 39 genes (63 genes total) that are well-established CPGs but for which RMS is not knowingly associated (eTable 1 in Supplement 1). Cancer-predisposition variants were defined as rare variants (minor allele frequency <0.01) that were either known pathogenic or likely pathogenic variants reported in ClinVar, version 20190305 (National Library of Medicine) or were novel loss-of-function variants (splicing, frameshift, or stop-gain variants).

### Statistical Analysis

The primary outcomes of the study were EFS, defined as time from the date of study enrollment to tumor recurrence or progression, second malignant neoplasm, or death due to any cause, and OS, defined as time from the study enrollment to death due to any cause. Patients without an event were censored at the time of the last contact. To evaluate the association of having a CPV with EFS and OS independent of a second malignant neoplasm, we also conducted a sensitivity analysis, excluding the 1 individual who had a CPV and developed a second malignant neoplasm.

We assessed differences in EFS and OS by CPV status in 24 RMS-associated CPGs and in the expanded list of 63 CPGs. Each analysis was also restricted to evaluate differences in EFS and OS by CPV status in patients with ERMS only because CPV frequency is significantly enriched in this subtype.^13,14^ To perform post hoc survival analyses stratified by *PAX3/7::FOXO1* fusion status, we coded all 347 patients with ERMS as fusion-negative because patients with ERMS almost always lack the canonical *PAX3/7::FOXO1* fusion gene.^15,16^ Only patients with ARMS with known *PAX3/7::FOXO1* fusion status (n = 80) were included in the fusion-stratified models. To account for multiple comparisons in our gene-group analysis, we applied a Bonferroni correction, which was based on evaluating 2 groups of gene sets in RMS overall and in ERMS (Bonferroni-corrected *P* < .01 was considered statistically significant). We also accounted for multiple comparisons in our gene-specific analyses. Specifically, we tested 4 genes for RMS overall and in ERMS (Bonferroni-corrected *P* < .007 was considered statistically significant). Therefore, we concluded statistical significance based on these corrected *P* values.

We generated survival curves and calculated survival probabilities across the 14-year study follow-up period using the Kaplan-Meier method; we also examined differences in outcome between patients with and without CPVs using the log-rank test. To control for the association of clinical covariates with outcome, we constructed multivariable Cox proportional hazards regression models to estimate the independent association of CPV status (CPV absent, CPV present) with EFS and OS using adjusted hazard ratios (AHRs), 95% CIs, and *P* values. The final model adjusted for age at diagnosis (categorical: <1 year, 1 to 9 years, and ≥10 years), tumor histology (ARMS, ERMS, or other), and tumor stage (categorical: I, II, III, or IV), variables that were statistically significantly (*P* < .05) associated with outcome in univariable models (eMethods in Supplement 1). We also included the first 5 principal components (derived from exome data^13^) to control for confounding by differences in allele frequency among underlying genetically similar subgroups.^17^ We did not include *PAX3/7::FOXO1* fusion status as a covariate in the multivariable models; this information was missing for 85% of the cohort because collecting data on histology, rather than fusion status (especially for patients with ERMS), was standard of care at the time of data collection. The threshold for statistical significance was set at 2-sided *P* < .05. All statistical analyses were conducted in R, version 4.04 (R Project for Statistical Computing). Data analysis was performed from June 16, 2021, to May 15, 2023.

## Results

### Study Population

There were 615 patients eligible for the study. We excluded 35 patients due to missing outcome and/or covariate data. Therefore, the final study cohort comprised 580 individuals, with a male-to-female ratio of 1.5 to 1 (229 female [39.5%] and 351 male [60.5%]). Demographic and clinical characteristics of the study cohort are summarized in Table 1. Median age at diagnosis was 5.9 years (range, 0.01-23.23 years), and the majority of patients were diagnosed with ERMS. In patients with ARMS with known fusion status (n = 80), 82.5% were fusion-positive RMS.

**Table 1.** Demographic and Clinical Characteristics of the Study Cohort^a^

Characteristic	All patients (n = 580)	RMS		Other or NOS (n = 66)	*P* value^b^
		Alveolar (n = 167)	Embryonal (n = 347)		
Sex					
Female	229 (39.5)	86 (51.5)	124 (35.7)	19 (28.8)	<.001
Male	351 (60.5)	81 (48.5)	223 (64.3)	47 (71.2)	
Age at diagnosis, y					
<1	38 (6.5)	15 (9.0)	20 (5.7)	3 (4.5)	<.001
1-9	352 (60.7)	74 (44.3)	240 (69.2)	38 (57.6)	
≥10	190 (32.8)	78 (46.7)	87 (25.0)	25 (37.9)	
Race and ethnicity					
Hispanic	86 (14.8)	34 (20.4)	43 (12.4)	9 (13.6)	.40
Non-Hispanic Black or African American	68 (11.7)	19 (11.4)	40 (11.5)	9 (13.6)	
Non-Hispanic White	348 (60.0)	94 (56.3)	214 (61.7)	40 (60.6)	
Other non-Hispanic racial or ethnic group^c^	33 (5.7)	8 (4.8)	21 (6.0)	4 (6.1)	
Unknown	45 (7.8)	12 (7.1)	29 (8.4)	4 (6.1)	
Primary tumor site					
Extremity	60 (10.3)	38 (22.8)	17 (4.9)	5 (7.6)	<.001
Genitourinary, bladder or prostate	48 (8.3)	2 (1.2)	40 (11.5)	6 (9.0)	
Genitourinary, nonbladder or nonprostate	86 (14.8)	4 (2.4)	74 (21.3)	8 (12.1)	
Head and neck	102 (17.6)	34 (20.4)	64 (18.4)	4 (6.1)	
Orbital	41 (7.0)	9 (5.4)	27 (7.8)	5 (7.6)	
Other	44 (7.6)	9 (5.4)	28 (8.1)	7 (10.6)	
Parameningeal	29 (5.0)	9 (5.4)	15 (4.3)	5 (7.6)	
Retroperitoneal or perineal	37 (6.4)	13 (7.8)	20 (5.8)	4 (6.1)	
Trunk	27 (4.7)	10 (6.0)	15 (4.3)	2 (3.0)	
Unknown	106 (18.3)	39 (23.4)	47 (13.6)	20 (30.3)	
Tumor stage					
I	179 (30.9)	21 (12.6)	135 (38.9)	23 (34.8)	<.001
II	81 (14.0)	30 (18.0)	47 (13.6)	4 (6.1)	
III	186 (32.0)	53 (31.7)	117 (33.7)	16 (24.3)	
IV	134 (23.1)	63 (37.7)	48 (13.8)	23 (34.8)	
CPV status^d^					
Absent	31 (5.3)	2 (1.2)	27 (7.8)	2 (3.0)	.003
Present	549 (94.7)	165 (98.8)	320 (92.2)	64 (97.0)	
Death					
No	393 (67.8)	77 (46.1)	268 (77.2)	48 (72.7)	<.001
Yes	187 (32.2)	90 (53.9)	79 (22.8)	18 (27.3)	
Event					
None	344 (59.3)	61 (36.5)	241 (69.5)	42 (63.6)	<.001
Relapse or progression	214 (36.9)	102 (61.1)	92 (26.5)	20 (30.3)	
Secondary malignant neoplasm	7 (1.2)	1 (0.6)	5 (1.4)	1 (1.5)	
Death without relapse or progression or secondary malignant neoplasm	15 (2.6)	3 (1.8)	9 (2.6)	3 (4.6)	

Abbreviations: CPV, cancer-predisposition variant; NOS, not otherwise specified; RMS, rhabdomyosarcoma.

^a^ Data are reported as number (percentage) of participants.

^b^ *P* values represent the significance of the association between each characteristic variable and histological subtype based on a χ^2^ or Fisher exact test.

^c^ Included individuals who self-identified as non-Hispanic Alaska Native, Asian, or Native Hawaiian or Pacific Islander.

^d^ Germline CPV in 1 of the 24 RMS-associated cancer-predisposition genes.

The median follow-up survival time of the study cohort was 4.75 years (IQR, 2.23-7.25 years). Of the 31 patients with a CPV in 1 of the 24 RMS-associated genes (eTable 2 in Supplement 1), 16 (51.6%) experienced an event: 12 experienced relapse or progression, of whom 10 died; 1 had a second malignant neoplasm and died; and 3 died of primary disease. Of the 549 patients without a CPV in an RMS-associated gene, 220 patients (40.1%) experienced an event: 202 experienced relapse or progression, of whom 161 died; 6 developed a second malignant neoplasm, of whom all survived; and 12 died of primary disease. Most patients who harbored a CPV and experienced an event had ERMS and carried a CPV in *TP53* or *HRAS*.

### RMS Outcome by CPV Status

**Figure 1.**
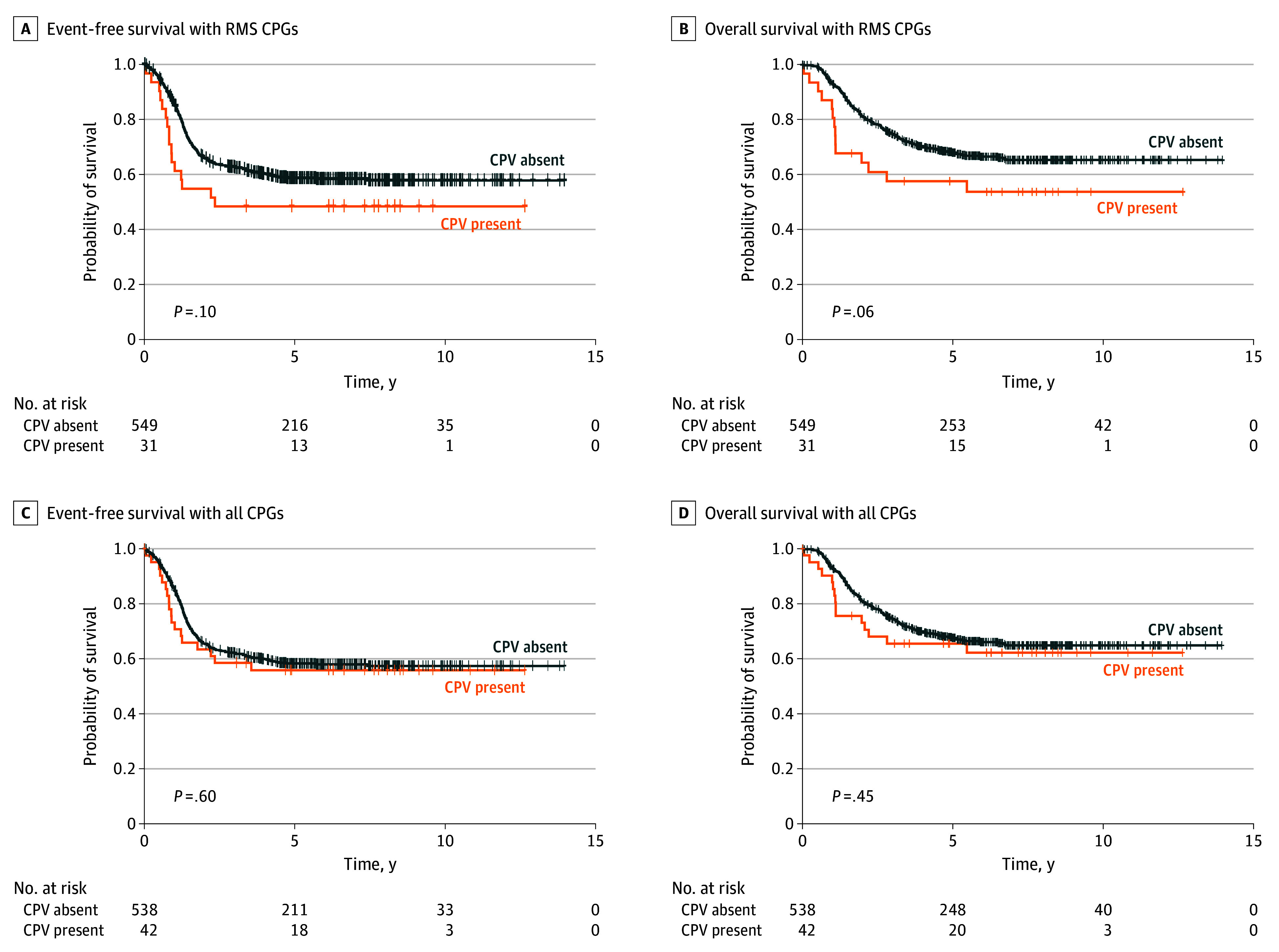
Kaplan-Meier Survival Outcomes of Cancer-Predisposition Variant (CPV) Status on Rhabdomyosarcoma (RMS) A and B, Survival of patients with RMS by the presence or absence of a CPV in any of the 24 RMS-associated cancer-predisposition genes (CPGs). C and D, Survival of patients with RMS by the presence or absence of a CPV in any of the 63 CPGs.

For patients with RMS who had a CPV in 1 of the 24 RMS-associated CPGs, EFS was 48.4% for patients with a CPV compared with 57.8% for patients without a CPV (*P* = .10) (Figure 1A). After adjusting for covariates, patients with a CPV in an RMS-associated gene had worse EFS compared with patients without a CPV in 1 of these genes (AHR, 2.01 [95% CI, 1.18-3.42]; *P* = .01) (Table 2), although this was not statistically significant. The OS was 53.7% for patients with a CPV in 1 of the RMS-associated CPGs compared with 65.3% for patients without a CPV (*P* = .06) (Figure 1B). After adjusting for covariates, patients with a CPV in an RMS-associated gene had significantly worse OS compared with patients without a CPV in 1 of these genes (AHR, 2.49 [95% CI, 1.39-4.45]; *P* = .002) (Table 2). In a sensitivity analysis that excluded the 1 individual with a second malignant neoplasm who harbored a CPV, we found that CPV status in an RMS-associated gene was still significantly associated with outcome, and the outcome estimates did not meaningfully change (<5% change in AHR).

**Table 2.** Multivariable Regression Models of Cancer-Predisposition Status on Outcome Among Individuals With RMS

Outcome	All patients			Embryonal RMS		
	No. of events	AHR (95% CI)^a^	*P* value^b^	No. of events	AHR (95% CI)^a^	*P* value^b^
**Event-free survival**						
RMS CPGs						
CPV absent	220	1 [Reference]	.01	92	1 [Reference]	.007
CPV present	16	2.01 (1.18-3.42)		14	2.25 (1.25-4.06)	
All CPGs						
CPV absent	218	1 [Reference]	.19	91	1 [Reference]	.06
CPV present	18	1.39 (0.85-2.28)		15	1.74 (0.98-3.07)	
**Overall survival**						
RMS CPGs						
CPV absent	173	1 [Reference]	.002	67	1 [Reference]	.002
CPV present	14	2.49 (1.39-4.45)		12	2.83 (1.47-5.43)	
All CPGs						
CPV absent	172	1 [Reference]	.05	65	1 [Reference]	.02
CPV present	15	1.71 (0.99-2.95)		12	2.15 (1.13-4.10)	

Abbreviations: AHR, adjusted hazard ratio; CPGs, cancer-predisposition gene; CPV, cancer-predisposition variant; RMS, rhabdomyosarcoma.

^a^ AHR for age at diagnosis, tumor stage, histological subtype (for all patient analyses), and the first 5 principal components.

^b^ Statistical significance was based on the Bonferroni-corrected *P* < 0.01.

When we expanded the analysis to patients with a CPV in any of the 63 CPGs, EFS was 55.9% compared with 57.4% for patients without a CPV (*P* = .60) (Figure 1C). The OS was 62.3% for patients with a CPV compared with 64.9% for patients without a CPV (*P* = .45) (Figure 1D). In adjusted models, there were no significant differences in EFS or OS by CPV status of the 63 CPGs (Table 2).

### RMS Outcome by CPV Status in Patients With ERMS

When we analyzed the outcome of patients with ERMS by CPV status in the RMS-associated CPGs, patients with a CPV had significantly worse EFS (AHR, 2.25 [95% CI, 1.25-4.06]; *P* = .007) and OS (AHR, 2.83 [95% CI, 1.47-5.43]; *P* = .002) compared with those without a CPV in 1 of these genes (Table 2). In our analysis of the expanded list of 63 CPGs, OS was significantly different between patients with ERMS with a CPV and those without a CPV (AHR, 2.15 [95% CI, 1.13-4.10]; *P* = .02) (Table 2).

### RMS Outcome by CPV Status in Specific Genes

We analyzed the association of CPVs in specific genes when there were at least 5 patients with RMS (which provided sufficient power for the analyses) with a CPV in a given gene: *TP53*, *NF1*, *HRAS*, and *BRCA2*.^13^ Among all RMS subtypes, we found significant differences in outcome by CPV status of *TP53* or *HRAS* but not for *NF1* or *BRCA2* (Table 3 and eFigure in Supplement 1). For EFS and OS, the greatest associations of *TP53* CPVs were observed in patients with ERMS (EFS: AHR, 4.43 [95% CI, 1.79-10.96]; *P* = .001; OS: AHR, 5.26 [95% CI, 1.92-14.41]; *P* = .001). Among all patients, we also found that those with a CPV in *HRAS* had worse EFS (AHR, 6.22 [95% CI, 2.23-17.41]; *P* < .001) and OS (AHR, 12.76 [95% CI, 4.44-36.67]; *P* < .001) compared with patients without a CPV in this gene.

**Table 3.** Multivariable Regression Models of Cancer-Predisposition Status by Gene on Outcome Among Individuals With RMS

Outcome	All patients			Embryonal RMS			
	No. of events	AHR (95% CI)^a^	*P* value^b^	No. of events		AHR (95% CI)^a^	*P* value^b^
**Event-free survival**							
*TP53*							
CPV absent	229	1 [Reference]	.002		100	1 [Reference]	.001
CPV present	7	3.42 (1.56-7.48)			6	4.43 (1.79-10.96)	
*HRAS*							
CPV absent	232	1 [Reference]	<.001		102	1 [Reference]	<.001
CPV present	4	6.22 (2.23-17.41)			4	6.10 (2.09-17.82)	
*NF1*							
CPV absent	234	1 [Reference]	.34		104	1 [Reference]	.40
CPV present	2	0.50 (0.12-2.05)			2	0.54 (0.13-2.24)	
*BRCA2*							
CPV absent	235	1 [Reference]	.20		106	1 [Reference]	NA
CPV present	1	0.28 (0.04-1.98)			0	NA	
**Overall survival**							
*TP53*							
CPV absent	181	1 [Reference]	.001		74	1 [Reference]	.001
CPV present	6	4.08 (1.73-9.62)			5	5.26 (1.92-14.41)	
*HRAS*							
CPV absent	183	1 [Reference]	<.001		75	1 [Reference]	<.001
CPV present	4	12.76 (4.44-36.67)			4	11.72 (3.78-36.30)	
*NF1*							
CPV absent	185	1 [Reference]	.53		77	1 [Reference]	.67
CPV present	2	0.63 (0.15-2.62)			2	0.73 (0.17-3.10)	
*BRCA2*							
CPV absent	186	1 [Reference]	.40		79	1 [Reference]	NA
CPV present	1	0.43 (0.06-3.11)			0	NA	

Abbreviations: AHR, adjusted hazard ratio; CPV, cancer-predisposition variant; NA, not applicable; RMS, rhabdomyosarcoma.

^a^ AHR for age at diagnosis, tumor stage, histological subtype (for all patient analyses), and the first 5 principal components.

^b^ Statistical significance was based on the Bonferroni-corrected *P* < 0.07.

### RMS Outcome Stratified by Fusion Status

**Figure 2.**
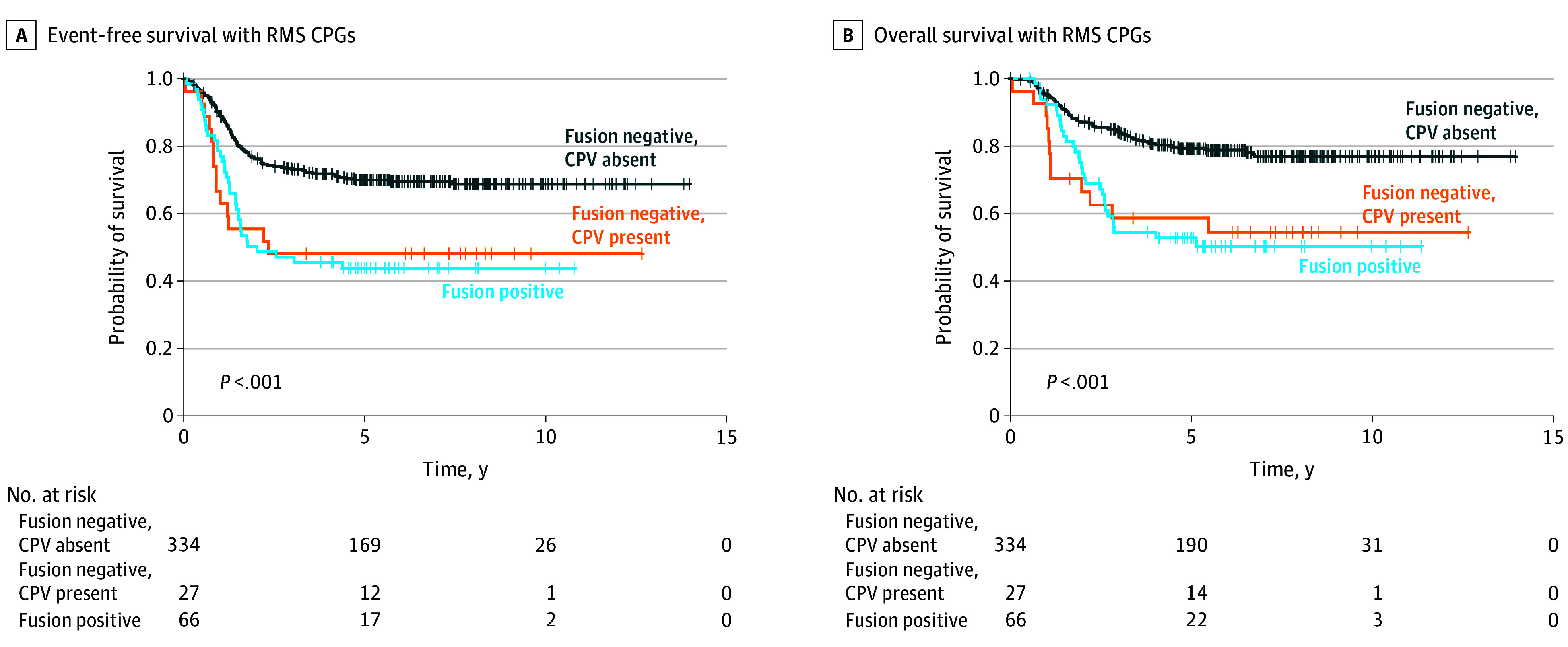
Kaplan-Meier Survival Outcomes of Cancer-Predisposition Variant (CPV) Status in Rhabdomyosarcoma (RMS)-Associated Cancer–Predisposition Genes (CPGs) by Fusion Status of *PAX3* or *PAX7* to the *FOXO1* Gene

Because the association of CPVs was greatest in the RMS-associated CPGs, we carried out a post hoc analysis of CPVs in those genes by *PAX3/7::FOXO1* fusion status. There were 427 patients with RMS for the analysis: 66 patients with fusion-positive RMS and 361 with fusion-negative RMS (we coded 347 patients with ERMS as fusion-negative RMS). We found that among patients who had fusion-negative RMS, those with a CPV had worse EFS (AHR, 2.28 [95% CI, 1.28-4.04]; *P* = .005) and OS (AHR, 2.80 [95% CI, 1.49-5.26]; *P* = .001) compared with those without a CPV (Figure 2). Compared with patients who had fusion-positive RMS, the outcome among patients who had fusion-negative RMS with a CPV was not significantly different for EFS (AHR, 1.35 [95% CI, 0.71-2.59]; *P* = .37) or OS (AHR, 1.71 [95% CI, 0.84-3.47]; *P* = .14).

## Discussion

In this cohort study, we found that germline CPVs in previously reported RMS-predisposition genes were significantly associated with EFS and OS. Additionally, associations on outcome were greatest for patients with ERMS histology. We also found that in analyses by *PAX3/7::FOXO1* fusion status, patients with fusion-negative RMS who harbored a CPV had comparable survival to those with fusion-positive RMS, the latter of which is associated with particularly aggressive tumors.

Children with cancer who harbor germline variants in *TP53* are more likely to develop second malignant neoplasms, often leading to poorer outcomes.^18^ Our study found, however, that second malignant neoplasms were not associated with RMS outcome by CPV status. This suggests that *TP53* variants were associated with outcome independent of second malignant neoplasms. Apart from the association of *TP53* with second malignant neoplasms, literature related to the impact of germline *TP53* variants on RMS outcome is limited. Shern et al^5^ found that individuals with fusion-negative RMS who harbored *TP53* somatic mutations had worse EFS compared with those with wild-type *TP53*. However, because the study lacked a matched germline sample, the authors^5^ also could not conclude whether the mutations were germline or somatic in origin. In contrast, our study did not have somatic data to examine associations of germline variants and subsequent somatic mutations in *TP53* or other loci. Thus, future work should aim to integrate both germline and somatic data to disentangle the mechanism by which these germline CPVs contribute to RMS etiology and outcome. Our findings could suggest that germline *TP53* variants were associated with poor treatment response and/or drug-resistant tumors. Casey et al^19^ found that *TP53* mutations were associated with RMS radioresistance. Thus, examining the association between *TP53* alterations and treatment response or drug resistance is an important avenue that could enhance knowledge for treatment decisions.

This is the first study, to our knowledge, to demonstrate that patients with RMS who harbor CPVs in *HRAS* have worse EFS and OS. All patients with RMS who harbored a CPV in *HRAS* had well-described variants that are known to cause Costello syndrome, an autosomal dominant disorder.^20^ However, only 1 of the 5 individuals in that study had a confirmed diagnosis of Costello syndrome. In individuals with Costello syndrome, diagnosis of RMS is typically associated with more favorable RMS survival outcomes.^20,21,22^ Our findings, however, suggest that individuals with RMS who had *HRAS* CPVs had a significantly greater risk of death than those without *HRAS* CPVs. As clinical inhibitors of RAS are being rapidly developed and used in the clinic,^23^ we hypothesize that children with RMS who have *HRAS* mutations may benefit from this more targeted therapeutic approach. However, since children with Costello syndrome have comorbidities that may impact outcome or the ability to deliver effective therapy, future studies should assess whether germline or somatic *HRAS* variants have selective effects on survival of individuals with RMS independent of Costello syndrome to determine how risk stratification may incorporate our findings.

Neither *NF1* nor *BRCA2* CPVs had a significant association with EFS or OS. A wide spectrum of pathogenic variants in *NF1* is associated with neurofibromatosis type 1, an autosomal-dominant disorder in which 0.5% to 1.0% of patients develop RMS.^24^ There is consensus that survival of these individuals is representative of those with non–neurofibromatosis type 1 ERMS,^24,25,26^ which agrees with our findings. Associations of germline *BRCA2* variants with RMS, to our knowledge, had not been previously reported before the identification of 6 individuals with *BRCA2* CPVs in Li et al.^13^ Our analysis suggests that these variants may not be significantly associated with EFS or OS among individuals with RMS. In the adult cancer literature, a study using data from The Cancer Genome Atlas on patients with ovarian cancer suggests that individuals with *BRCA2* variants may have a better outcome due to higher sensitivity to chemotherapy compared with individuals with wild-type genotypes.^27^

As noted, *PAX3/7::FOXO1* fusion status is a strong predictor of RMS outcome.^12^ We found that among patients with fusion-negative RMS, those with CPVs in RMS-associated CPGs were more likely to have worse survival than those without CPVs in these genes. We also found that the outcome among patients who had fusion-negative RMS with a CPV was not significantly different than patients who had fusion-positive RMS; the outcome was worse compared with patients with fusion-positive RMS, although this association was not statistically significant. Because the fusion-stratified analysis was limited by sample size, a larger sample of patients who had fusion-positive RMS is needed to validate these results.^28^ In the present study, none of the patients with fusion-positive RMS harbored a CPV.^13^ Because survival was comparable to patients with fusion-positive RMS, CPV status among patients with fusion-negative RMS might represent an important prognostic factor for this group of patients.

Our overall findings contrast with a study by Kim et al,^14^ which evaluated EFS in 256 patients with RMS. The authors found no significant associations between EFS and pathogenic or likely pathogenic variants in 130 cancer-susceptibility genes, even after adjusting for significant covariates. Differences in results are likely because our analysis focused on a smaller number of specific RMS-associated genes, which could have circumvented issues related to signal dilution. Other reasons include sample size, as our study had more than twice the number of patients. Additionally, Kim et al^14^ focused on evaluating the outcome among 256 patients with intermediate risk disease from the COG ARST0531 clinical trial, while our study population was an unselected group of patients with RMS.

### Strengths and Limitations

Strengths of the current study include assessment of an unselected cohort of patients with RMS, representative of population-based cohorts with RMS. Furthermore, we evaluated extensive clinical information on one of the largest cohorts of patients with RMS, and we present analyses of the recently revised diagnostic risk stratification criteria, which include fusion status.

One limitation of this work is that half of the patients with ARMS did not undergo *PAX3/7::FOXO1* fusion testing, which limited the number of patients who were included in the post hoc analyses. However, of the patients with known FOXO1 fusion status, 82.5% of them had fusion-positive RMS, which is consistent with previous estimates.^12^ We also recognize that studies have shown that individuals with fusion-negative *MYOD1*-associated sclerosing or spindle cell RMS have a poor prognosis.^29^ However, we were unable to evaluate survival differences for this subgroup due to a lack of data on somatic *MYOD1* status. Additionally, although we found that second malignant neoplasms were not associated with RMS outcome by CPV status, we also acknowledge that further follow-up beyond 5 years is needed to fully consider the impact of second malignant neoplasms. It should also be noted that we did not include a treatment protocol as a covariate in the multivariable analyses, as recent RMS clinical trials suggest no differences in survival according to these protocols.^8,12,30^

## Conclusions

This cohort study demonstrated that patients with germline CPVs in RMS-associated CPGs, especially *TP53* and *HRAS*, had a significantly worse outcome compared with those without these CPVs, findings not driven by secondary malignant neoplasms. Currently, germline genetic testing is not routinely performed at diagnosis of RMS to determine risk status, suggesting that germline testing for RMS-associated CPGs should be considered in clinical settings for both prognosis and subsequent cancer surveillance, including cascade testing of family members to initiate tumor surveillance. The study’s findings, that patients with fusion-negative RMS who harbored a CPV had comparable survival to those with fusion-positive RMS, also suggest that COG diagnostic risk stratification should consider the incorporation of CPV status in prospective risk-based clinical trials.

## Notes

Supplement 1.
eMethods.
**eTable 1.** Cancer Predisposition Genes as Evaluated in Li et al **eTable 2.** Outcome of Individuals With Rhabdomyosarcoma who Harbored Cancer-Predisposition Variants **eFigure.** Cox Proportional Hazards Regression Models of Cancer Predisposition Variant (CPV) Status by Specific Genes

Supplement 2.
Data Sharing Statement
