# Supplementary material for: Germline Genetic Testing and Survival Outcomes Among Children With Rhabdomyosarcoma: A Report From the Children’s Oncology Group
Source: JAMA Netw Open. 2024 Mar 28;7(3):e244170. doi: 10.1001/jamanetworkopen.2024.4170 (PMC10979319; doi:10.1001/jamanetworkopen.2024.4170)
Supplement: Supplement 1. — eMethods. eTable 1. Cancer Predisposition Genes as Evaluated in Li et al eTable 2. Outcome of Individuals With Rhabdomyosarcoma who Harbored Cancer-Predisposition Variants eFigure. Cox Proportional Hazards Regression Models of Cancer Predisposition Variant (CPV) Status by Specific Genes [file jamanetwopen-e244170-s001.pdf]

Supplemental Online Content

Martin-Giacalone BA, Li H, Scheurer ME, et al. Germline genetic testing and survival outcomes among children with rhabdomyosarcoma: a report from the Children's Oncology Group. *JAMA Netw Open*. 2024;7(3):e244170.
doi:10.1001/jamanetworkopen.2024.4170

eMethods

eTable 1. Cancer Predisposition Genes as Evaluated in Li et al

eTable 2. Outcome of Individuals With Rhabdomyosarcoma who Harbored Cancer-Predisposition Variants

eFigure. Cox Proportional Hazards Regression Models of Cancer Predisposition Variant (CPV) Status by Specific Genes

This supplemental material has been provided by the authors to give readers additional information about their work.

© 2024 Martin-Giacalone BA, et al. *JAMA Network Open*.

eMethods

Population

Demographic, phenotypic, and outcome data were collected through the COG D9902 soft tissue sarcoma biobanking protocol. COG central pathology review was required for individuals enrolled on front-line COG RMS therapeutic study; in cases where patients were not planning to be enrolled on a RMS therapeutic study, the enrolling institution made the histological diagnosis. Data collected by self-report included sex (female, male), race (American Indian/Alaska Native, Asian, Black or African American, Native Hawaiian/Pacific Islander, White, unknown or not reported), and ethnicity (Hispanic, Not Hispanic, or unknown or not reported). These data were collected to assess the association of demographic characteristics with the outcome of interest. Clinical data on age at diagnosis (continuous), stage (1, 2, 3, 4), histological subtype (alveolar, botryoid, embryonal, mixed RMS, RMS not otherwise specified, RMS with ganglionic differentiation, spindle cell), primary site, and PAX3/7::FOXO1 fusion status (fusion-positive RMS, fusion-negative RMS, unknown) were collected by the clinical trial investigator. For this study, we recoded case histology that was not ERMS or ARMS; botryoid (n=38) or spindle cell RMS (n=30) were coded as ERMS, while mixed RMS, RMS with ganglionic differentiation, and RMS not otherwise specified were coded as "other."

Of the 615 patients enrolled in the initial study, 300 patients were co-enrolled on one or more of the following COG therapeutic studies: ARST0921, ARST08P1, ARST0531, ARST0431, ARST0331, D9803, D9802, and D9602.

Statistical analysis

In multivariable Cox proportional hazards regression, covariates of interest included: age at diagnosis (categorical variable with groups <1, 1-9, and ≥10 years), sex assigned at birth (male, female), primary site, tumor histology (ARMS, ERMS, and other), tumor stage (categorical variable with groups 1, 2, 3, and 4; includes primary site, tumor size, regional nodal status, and presence of metastases), and the top five principal components (to control for population stratification), which were derived from exome data. We tested all covariates in a backwards, stepwise selection method to determine variables that were significantly associated with RMS outcome at the $P<0.05$ level. Based on our hypothesis, the initial model included CPV status and allowed for the addition of all covariates. We removed the variables sex assigned at birth and primary site from the multivariable model as they did not significantly change the effect estimate upon addition/removal from the model. Confidence intervals were estimated using the log-log survival function. All Cox models met the proportional hazards assumptions based on assessing Schoenfeld residuals or a time transformation ($\alpha=0.05$).

© 2024 Martin-Giacalone BA, et al. *JAMA Network Open*.

eTable 1. Cancer Predisposition Genes as Evaluated in Li et al.[a]

RMS predisposition genes (n=24)	Additional cancer predisposition genes (n=39)
BRAF	*ALK*
CBL	*APC*
CDKN1C	*BAP1*
CHEK2	*BMPR1A*
CREBBP	*BRCA1*
DICER1	*BRCA2*
HRAS	*CDC73*
KRAS	*CDH1*
MAP2K1	*CDK4*
MAP2K2	*CDKN2A*
MLH1	*CEBPA*
MSH2	*EPCAM*
MSH6	*FH*
NF1	*GATA2*
NRAS	*MAX*
PMS2	*MEN1*
PTCH1	*NF2*
PTPN11	*PALB2*
RAF1	*PAX5*
RIT1	*PHOX2B*
SHOC2	*PRKAR1A*
SOS1	*PTEN*
SUFU	*RB1*
TP53	*RET*
	RUNX1
	SDHA
	SDHAF2
	SDHB
	SDHC
	SDHD
	SMAD4
	SMARCA4
	SMARCB1
	STK11
	TMEM127
	TSC1
	TSC2
	VHL
	WT1

[a] Li H, Sisoudiya SD, Martin-Giacalone BA, et al: Germline Cancer Predisposition Variants in Pediatric Rhabdomyosarcoma: A Report From the Children’s Oncology Group. J Natl Cancer Inst 113:875–883, 2021

© 2024 Martin-Giacalone BA, et al. *JAMA Network Open*.

eTable 2. Outcome of Individuals With Rhabdomyosarcoma Who Harbored Cancer-Predisposition Variants

Gene and cancer-predisposition variant	ClinVar status	ClinVar Variation ID	Patient tumor histology	Patient age at diagnosis (years)	Sex	Primary site	Event type
RMS-associated cancer-predisposition genes							
TP53 (ENST00000269305.4)							
c.892G>T (p.Glu298Ter)	P	93323	ERMS	1-9	Male	Arm	Relapse/Progression, Death
c.818G>A (p.Arg273His)	P	12366	ERMS	1-9	Female	Thigh	No Event
c.794T>C (p.Leu265Pro)	P/LP	245777	ARMS	1-9	Male	Nasopharynx (Parameningeal)	Death
c.743G>A (p.Arg248Gln)	P	12356	ERMS	<1	Male	Paraspinal	No Event
c.730G>A (p.Gly244Ser)	P	376600	ERMS	1-9	Female	Middle Ear	Relapse/Progression, Death
c.560-1G>C	P	492748	ERMS	1-9	Female	Thigh	Relapse/Progression
c.473G>A (p.Arg158His)	P/LP	141963	Other	1-9	Female	Unknown	No Event
c.451C>T (p.Pro151Ser)	P/LP	12370	ERMS	<1	Female	Scalp	No Event
c.451C>A (p.Pro151Thr)	P/LP	12369	ERMS	1-9	Male	Unknown	Relapse/Progression, Death
c.375G>A (p.Thr125=)	P	177825	ERMS	1-9	Male	Thigh	Second Malignancy, Death
c.365_366del (p.Val122AspfsTer26)	P	127809	ERMS	<1	Female	Bladder	Relapse/Progression, Death
NF1 (NM_001042492.3)							
c.1466A>G (p.Tyr489Cys)	P	354	ERMS	1-9	Female	Peritoneum	No Event
c.2041C>T (p.Arg681Ter)	P	188280	ERMS	1-9	Female	Vagina	No Event
c.3520C>T (p.Gln1174Ter)	P	978806	ERMS	1-9	Male	Orbit	No Event
c.3826C>T (p.Arg1276Ter)	P	237556	ERMS	≥10	Female	Cervix	Relapse/Progression, Death
c.4985G>A (p.Trp1662Ter)	P	573015	ERMS	1-9	Male	Testis-Paratestis	No Event

© 2024 Martin-Giacalone BA, et al. *JAMA Network Open*.

Gene and cancer-predisposition variant	ClinVar status	ClinVar Variation ID	Patient tumor histology	Patient age at diagnosis (years)	Sex	Primary site	Event type
NF1 (NM_001042492.3) continued							
c.5305C>T (p.Arg1769Ter)	P	228381	ERMS	<1	Male	Shoulder girdle	No Event
c.5609G>A (p.Arg1870Gln)	P	185354	ERMS	1-9	Male	Pelvis, Site Indeterminate	No Event
c.6704+1G>T	P	547680	ERMS	1-9	Male	Testis-Paratestis	No Event
c.7159_7164del (p.Asn2387_Phe2388del)	P/LP	220715	ERMS	1-9	Female	Pelvis, site indeterminate	Relapse/Progression, Death
HRAS (NM_005343.4)							
c.35G>C (p.Gly12Ala)	P	12603	ERMS	1-9	Female	Unknown	Relapse/Progression, Death
c.34G>A (p.Gly12Ser)	P	12602	ERMS	1-9	Male	Pelvis, Site Indeterminate	Relapse/Progression, Death
c.34G>A (p.Gly12Ser)	P	12602	ERMS	1-9	Male	Orbit	No Event
c.34G>A (p.Gly12Ser)	P	12602	ERMS	1-9	Female	Pelvis, site indeterminate	Relapse/Progression, Death
c.34G>A (p.Gly12Ser)	P	12602	ERMS	1-9	Male	Abdominal wall	Death
CBL (NM_005188.4):							
c.1259G>A (p.Arg420Gln)	P/LP	13810	ERMS	1-9	Female	Retroperitoneum	Relapse/Progression
c.1495C>T (p.Arg499Ter)	-	-	ERMS	1-9	Female	Retroperitoneum	No Event
DICER1 (NM_030621.4)							
c.2026C>T (p.Arg676Ter)	P	242054	ERMS	1-9	Female	Eye	No event
MSH2 (NM_000251.3)							
c.1147C>T (p.Arg383Ter)	P	90554	ARMS	≥10	Female	Unknown	Death
PMS2 (NM_000535.7)							
c.2404C>T (p.Arg802Ter)	P	9237	ERMS	1-9	Female	Cheek	Relapse/Progression, Death

© 2024 Martin-Giacalone BA, et al. *JAMA Network Open*.

Gene and cancer-predisposition variant	ClinVar status	ClinVar Variation ID	Patient tumor histology	Patient age at diagnosis (years)	Sex	Primary site	Event type
PTCH1 (NM_001083602.3)							
c.93C>G	LP	978808	Other	1-9	Male	Pelvis, site indeterminate	No event
		Other cancer-predisposition genes					
BRCA2 (NM_000059.4)							
c.462_463del (p.Asp156Ter)	P	51684	ERMS	<1	Female	Bladder	No Event
c.3103G>T (p.Glu1035Ter)	P	51400	ERMS	1-9	Male	Retroperitoneum	No Event
c.3599_3600del (p.Cys1200Ter)	P	51493	ERMS	1-9	Female	Orbit	No Event
c.3847_3848del (p.Val1283LysfsTer2)	P	37859	ARMS	≥10	Male	Paranasal Sinus	Relapse/Progression, Death
c.7133C>G (p.Ser2378Ter)	P	38085	ERMS	1-9	Male	Middle Ear	No Event
c.7857G>A (p.Trp2619Ter)	P	38122	ARMS	1-9	Female	Unknown	No Event
SDHA (NM_004168.4)							
c.2T>G (p.Met1Arg)	P/LP	422382	ERMS	1-9	Male	Testis-Paratestis	No event
c.91C>T (p.Arg31Ter)	P/LP	142601	ARMS	≥10	Female	Unknown	No event
ALK (NM_004304.5)							
c.4297del (p.Glu1433ArgfsTer44)	VUS	1346971	ERMS	1-9	Male	Other head & neck	Relapse/Progression
BRCA1 (NM_007300.4)							
c.5240_5243del (p.Arg1747LysfsTer3)	P	37644	ERMS	1-9	Female	Other head & neck	No event
SDHC (NM_003001.5)							
c.386G>A (p.Trp129Ter)	P	978229	ERMS	1-9	Male	Testis-Paratestis	No event

[a] Variants are reported using genome assembly GRCh37/hg19

[b] P/LP, pathogenic or likely pathogenic

© 2024 Martin-Giacalone BA, et al. *JAMA Network Open*.

[c] VUS, variant of uncertain significance

[d] ERMS, embryonal rhabdomyosarcoma; ARMS, alveolar rhabdomyosarcoma

[e] Median follow-up time for individuals with a cancer predisposition variant was 4.88 years (interquartile range: 1.24-7.56 years)

eFigure. Cox Proportional Hazards Regression Models of Cancer Predisposition Variant (CPV) Status by Specific Genes

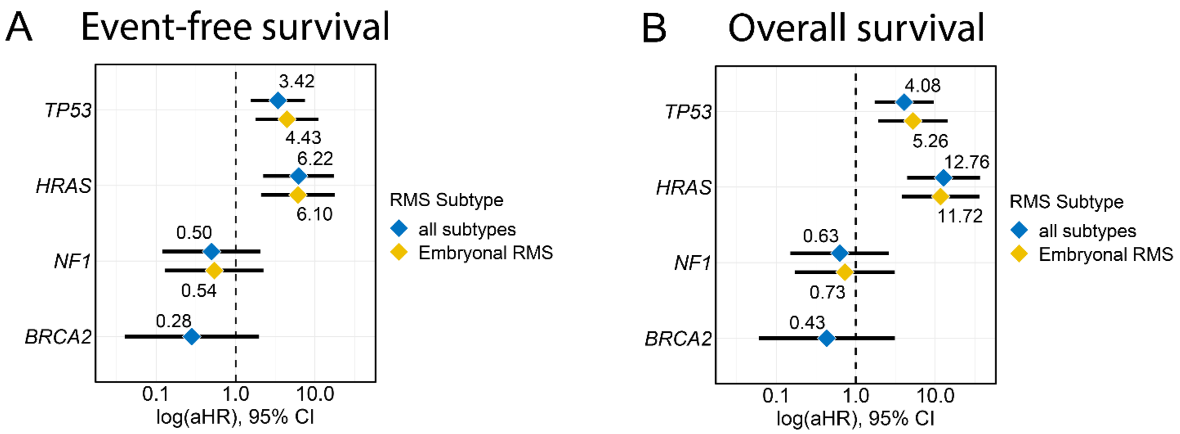

Adjusted hazard ratios (aHR) and 95% confidence intervals for RMS (A) event-free survival and (B) overall survival are plotted by specific genes and subtype group. RMS subtype group included either all subtypes (alveolar, embryonal, and other/not otherwise specified) or only embryonal RMS (ERMS) cases. No individuals with ERMS carried a CPV in BRCA2. Plot labels for aHR reflect the original aHR values, while the x-axis scale represents the log of the aHR.

© 2024 Martin-Giacalone BA, et al. *JAMA Network Open*.
