# Supplementary material for: Germline Genetic Testing and Survival Outcomes Among Children With Rhabdomyosarcoma: A Report From the Children’s Oncology Group
Source: JAMA Netw Open. 2024 Mar 28;7(3):e244170. doi: 10.1001/jamanetworkopen.2024.4170 (PMC10979319; doi:10.1001/jamanetworkopen.2024.4170)
Supplement: Supplement 2. — Data Sharing Statement [file jamanetwopen-e244170-s002.pdf]

# Data Sharing Statement

Martin-Giacalone. Germline Genetic Testing and Survival Outcomes Among Children With Rhabdomyosarcoma. *JAMA Netw Open*. Published March 28, 2024.
doi:10.1001/jamanetworkopen.2024.4170

**Data**
**Data available:** Yes
**Data types:** Deidentified participant data
**How to access data:** https://www.ncbi.nlm.nih.gov/projects/gap/cgi-bin/study.cgi?study_id=phs003192.v1.p1
**When available:** With publication

**Supporting Documents**
**Document types:** None

**Additional Information**
**Who can access the data:** Researchers whose proposed use of the data has been approved.
**Types of analyses:** Genetic analyses.
**Mechanisms of data availability:** Data are available through dbGaP.
